# 10-Year Impact of Transcatheter Aortic Valve Replacement Leaflet Design (Intra- Versus Supra-Annular) in Mortality and Hemodynamic Performance

**DOI:** 10.3389/fcvm.2022.924958

**Published:** 2022-06-08

**Authors:** Andrea Scotti, Luca Nai Fovino, Augustin Coisne, Tommaso Fabris, Francesco Cardaioli, Mauro Massussi, Giulio Rodinò, Alberto Barolo, Mauro Boiago, Saverio Continisio, Carolina Montonati, Tommaso Sciarretta, Vittorio Zuccarelli, Valentina Bernardini, Giulia Masiero, Massimo Napodano, Chiara Fraccaro, Alfredo Marchese, Giovanni Esposito, Juan F. Granada, Azeem Latib, Sabino Iliceto, Giuseppe Tarantini

**Affiliations:** ^1^Montefiore Einstein Center for Heart and Vascular Care, Montefiore Medical Center, Albert Einstein College of Medicine, Bronx, NY, United States; ^2^Cardiovascular Research Foundation, New York, NY, United States; ^3^Department of Cardiac, Thoracic, Vascular Sciences and Public Health, University of Padua Medical School, Padua, Italy; ^4^Unit of Cardiology, GVM Care and Research, Anthea Hospital, Bari, Italy; ^5^Department of Advanced Biomedical Sciences, Divisions of Cardiology and Cardiothoracic Surgery, University of Naples Federico II, Naples, Italy

**Keywords:** transcatheter aortic valve replacement, intra-annular, supra-annular, bioprosthetic valve failure, hemodynamic valve deterioration

## Abstract

**Background:**

The impact of transcatheter aortic valve replacement (TAVR) leaflet design on long-term device performance is still unknown. This study sought to compare the clinical and hemodynamic outcomes of intra- (IA) versus supra-annular (SA) TAVR designs up-to 10-years following implantation.

**Methods:**

Consecutive patients with at least 5-years follow-up following TAVR for severe symptomatic aortic stenosis from June 2007 to December 2016 were included. Bioprosthetic valve failure (BVF) and hemodynamic valve deterioration (HVD) were defined according to VARC-3 updated definitions and estimated using cumulative incidence function to account for the competing risk of death.

**Results:**

A total of 604 patients (82 years; 53% female) were analyzed and divided into IA (482) and SA (122) groups. Overall survival rates at 10-years were similar (IA 15%, 95%CI: 10–22; SA 11%, 95%CI: 6–20; *p* = 0.21). Compared to the SA TAVR, mean transaortic gradients were significantly higher and increased over time in the IA group. IA TAVRs showed higher 10-year cumulative incidences of BVF (IA 8% vs. SA 1%, *p* = 0.02) and severe HVD (IA 5% vs. SA 1%, *p* = 0.05). The occurrence of BVF and HVD in the IA group occurred primarily in the smallest TAVR devices (20–23-mm). After excluding these sizes, the cumulative incidences of BVF (IA 5% vs. SA 1%, *p* = 0.40) and severe HVD (IA 2% vs. SA 1%, *p* = 0.11) were similar.

**Conclusion:**

In this study, TAVR leaflet design had no impact on survival at 10-years. IA devices showed higher transaortic gradients and cumulative incidences of HVD and BVF predominantly occurring in the smallest valve sizes.

## Graphical Abstract

10-year outcomes and hemodynamic performance of intra-annular vs. supra-annular TAVR. HVD, hemodynamic valve deterioration; TAVR, transcatheter aortic valve replacement; VARC, valve academic research consortium. 
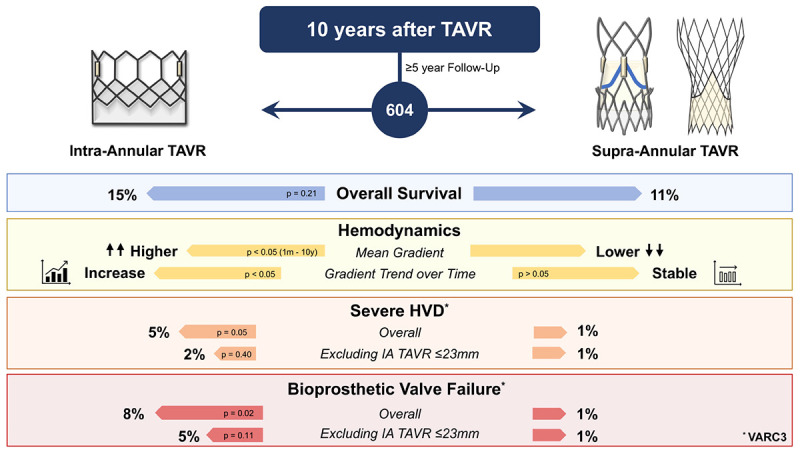

## Introduction

Transcatheter aortic valve replacement (TAVR) is an established treatment option for patients with symptomatic severe aortic stenosis. Given that TAVR is now being considered as a valid alternative to surgery even for low-risk patients, its indication is rapidly extending to younger patients having longer life expectancy (1, 2). Then, understanding TAVR durability and lifetime management is becoming a key aspect of patient care (3).

Transcatheter aortic valve replacement technologies can be broadly classified by the location of the leaflets into intra-annular (IA) or supra-annular (SA) designs. Randomized controlled trials have shown comparable outcomes and hemodynamic performance of these two designs compared to surgical bioprostheses up to 5 years (4–6). Clinical data suggest that surgical bioprostheses failure starts to occur and continues to increase after 5 years post-procedure (7). TAVR data beyond 5 years is limited by small sample sizes, and do not examine the impact of design in clinical outcomes and hemodynamic performance.

In this study, we aimed to determine the frequency of bioprosthetic valve failure (BVF) and hemodynamic valve deterioration (HVD) in IA vs. SA devices up to 10 years after TAVR, defined according to the new Valve Academic Research Consortium (VARC) 3 criteria (8).

## Materials and Methods

### Study Population

The study population consisted of consecutive patients undergoing TAVR, whose data were prospectively collected since June 2007 in the Padua University REVALVing Experience (PUREVALVE) registry. For the purpose of this analysis, we excluded patients with (a) less than 5-year of follow-up (inclusion period: June 2007–December 2016; *n* = 33), (b) not meeting the VARC-3 technical success criterion (alive, Successful access and delivery of the device, successful retrieval of the delivery system, correct positioning of a single prosthetic heart valve into the proper anatomical location, and freedom from surgery or intervention related to the device or to a major vascular or access-related, or cardiac structural complication; *n* = 5), (c) those treated for bicuspid aortic stenosis (*n* = 7), and (d) patients undergoing valve-in-valve procedures for degenerated surgical or transcatheter bioprostheses (*n* = 8). As such, 604 patients were deemed eligible to be included in this study. Indications for TAVR and vascular approach were based on Heart Team decision. All patients provided written informed consent for the procedure and data collection. The study was approved by the institutional Ethics Committee and conforms to the principles outlined in the Declaration of Helsinki.

### Device and Procedure

Four types of TAVR devices were implanted in this cohort of patients. The IA devices were the balloon-expandable Sapien/-XT/-3 (Edwards Lifesciences, Irvine, CA, United States), and the mechanically expandable LOTUS (Boston Scientifics, Marlborough, MA, United States). The SA devices were the self-expanding CoreValve/Evolut R (Medtronic, Minneapolis, MN, United States), and the self-expanding Acurate (Boston Scientifics, Marlborough, MA, United States). The choice and sizing of the bioprosthesis were based on multidetector computed tomography evaluation or, when this was not available (28 patients, years: 2007–2008), by integration of echocardiography, angiography, and/or simultaneous aortography during balloon valvuloplasty. In the absence of recent coronary intervention, discharge therapy consisted of 3–6 months of dual antiplatelet therapy, or the combination of oral anticoagulant and aspirin if anticoagulation was clinically indicated. Clinical and echocardiographic follow-up was routinely performed at 30 days, 6 months, 1 year, and yearly thereafter.

### Endpoints

The primary endpoints of the study were the incidences of BVF, moderate and severe hemodynamic valve deterioration (HVD), and all-cause mortality.

The new VARC-3 criteria were adopted for event adjudication (8). Moderate HVD was defined with an increase in mean transvalvular gradient ≥ 10 mmHg resulting in mean gradient ≥ 20 mmHg with concomitant decrease in effective orifice area ≥ 0.3 cm^2^ or ≥ 25% and/or decrease in Doppler velocity index ≥ 0.1 or ≥ 20% compared with echocardiographic assessment performed 1–3 months post-procedure, OR new occurrence or increase of ≥ 1 grade of intra-prosthetic aortic regurgitation (AR) resulting in ≥ moderate AR. Severe HVD was defined as an increase in mean transvalvular gradient ≥ 20 mmHg resulting in mean gradient ≥ 30 mmHg with concomitant decrease in effective orifice area ≥ 0.6 cm^2^ or ≥ 50% and/or decrease in Doppler velocity index ≥ 0.2 or ≥ 40% compared with echocardiographic assessment performed 1–3 months post-procedure, OR new occurrence, or increase of ≥ 2 grades, of intra-prosthetic AR resulting in severe AR. BVF was adjudicated in case of valve-related death, aortic valve re-operation or reintervention, and/or any bioprosthetic valve dysfunction associated with clinically expressive criteria (new-onset or worsening symptoms, left ventricular dilation/hypertrophy/dysfunction, or pulmonary hypertension) or irreversible severe HVD.

### Statistical Analysis

Descriptive statistics of continuous variables are reported as mean ± standard deviation or median [interquartile range (IQR)] and compared with the Student’s unpaired *t*-test (parametric test) or the Wilcoxon rank-sum test (non-parametric test), according to their distribution. Categorical variables were reported as absolute and relative frequencies and compared with the χ^2^ test with Yates’ correction for continuity or the Fisher exact test as appropriate. Survival curves with their 95% confidence interval (CI) were plotted using the Kaplan Meier estimator and compared with the log-rank test. The incidences of HVD and BVF were estimated using the cumulative incidence function (CIF) accounting for death as a competing risk. Mean transaortic gradients collected throughout the overall follow-up have been reported as means and medians [IQR] per each time point and displayed with the appropriate graphical presentations. Pairwise comparisons between IA and SA TAVRs, were adjusted using the Bonferroni multiple testing correction method. Paired analyses between every time point and the post-procedural echocardiographic evaluation were performed using the Wilcoxon signed rank-sum test. As a sensitivity analysis, the same comparison is provided with the use of the Student’s paired *t*-test. To avoid bias due to complete case analyses, missing data in baseline characteristics were handled with Multivariate Imputation via Chained Equations using the mice package (v3.13.0; van Buuren & Groothuis-Oudshoorn, 2011). For all analyses, a two-sided *p* < 0.05 was considered to be significant. Statistical analyses were performed using R, version 4.0.2 (R Foundation), packages cmprsk, survival.

## Results

### Baseline and Procedural Characteristics

A total of 604 TAVR patients from the PUREVALVE registry with a minimum follow-up of at least 5 years were eligible for the study. The population was stratified according to the IA (*n* = 482) or SA (*n* = 122) leaflet design, whose models and sizes are reported in Supplementary Table 1. In the IA group, all the valves were either Sapien/-XT/-3 (88%) or Lotus (12%). In the SA group, implanted valves were either CoreValve/Evolut R (81%) or Acurate (19%). Baseline clinical, echocardiographic, and procedural characteristics of the study population are summarized in Table 1. The overall cohort presented a median age of 82 years, 53% were females, and the median STS score was 4.8 [IQR: 3.1, 10.2]. Patients receiving SA TAVR had a more frequent history of previous myocardial infarction (26% vs. 16%, *p* = 0.018), coronary artery bypass surgery (18% vs. 11%, *p* = 0.05), chronic kidney disease (60% vs. 46%, *p* = 0.009), and presented a slightly lower left ventricular ejection fraction (53 ± 13 vs. 56 ± 12, *p* = 0.029). The majority of procedures were performed under local anesthesia and through femoral access, with increased use of pre- (81% vs. 67%, *p* = 0.003) and post-dilatation (36% vs. 8%, *p* < 0.001) of the bioprosthetic valve in the SA cohort.

**TABLE 1:** Baseline characteristics.

Clinical characteristics	Total (604)	Intra-annular (482)	Supra-annular (122)	*p*-value
Age, years	82 (78, 85)	82 (78, 85)	81 (78, 85)	0.953
Female	321 (53)	255 (53)	66 (54)	0.893
BMI	26 (24, 29)	26 (24, 290	26 (23, 28)	0.429
Hypertension	543 (91)	436 (91)	107 (88)	0.404
Diabetes mellitus	175 (29)	145 (30)	30 (25)	0.279
Dyslipidemia	377 (62)	310 (64)	67 (55)	0.070
Atrial fibrillation	195 (33)	160 (34)	35 (29)	0.390
Coronary artery disease	345 (58)	266 (56)	79 (65)	0.073
Previous myocardial infarction	111 (18)	79 (16)	32 (26)	0.018
Previous PCI	198 (33)	154 (32)	44 (36)	0.449
Previous CABG	75 (12)	53 (11)	22 (18)	0.050
Previous cardiac surgery	93 (15)	69 (14)	24 (20)	0.185
Previous stroke	78 (13)	58 (12)	20 (16)	0.258
COPD	151 (25)	125 (26)	26 (21)	0.349
Chronic kidney disease*	295 (49)	222 (46)	73 (60)	0.009
Prior pacemaker	51 (8)	43 (9)	8 (7)	0.511
NYHA class III/IV	339 (56)	262 (54)	77 (63)	0.101
STS score	4.8 (3.1, 10.2)	4.6 (3.1–10.2)	5.2 (3.2, 10)	0.537
**Echocardiographic data**				
Mean aortic gradient, mmHg	44 ± 15	44 ± 15	45 ± 16	0.950
Aortic valve area, cm^2^	0.79 ± 0.22	0.79 ± 0.22	0.81 ± 0.24	0.380
Indexed aortic valve area, cm^2^/m^2^	0.45 ± 0.13	0.45 ± 0.12	0.47 ± 0.14	0.129
LVEF, %	55 ± 12	56 ± 12	53 ± 13	0.029
**Procedural data**				
Anesthesia				0.879
Local	395 (65)	314 (65)	81 (67)	
General	209 (35)	168 (35)	41 (33)	
Access				<0.001
*Trans-*femoral	407 (68)	314 (66)	93 (77)	
*Trans-*subclavian	5 (1)	0 (0)	5 (4)	
*Trans-*apical	179 (30)	156 (33)	23 (19)	
*Trans-*aortic	7 (1)	7 (2)	0 (0)	
Pre-dilatation	420 (70)	321 (67)	99 (81)	0.003
Post-dilatation	84 (14)	40 (8)	44 (36)	<0.001

*defined as estimated glomerular filtration rate < 60 mL/min. BMI, body mass index; CABG, coronary artery bypass grafting; COPD, chronic obstructive pulmonary disease; LVEF, left ventricular ejection fraction; NYHA, New York Heart Association; PCI, Percutaneous Coronary Intervention; STS, Society of Thoracic Surgeons.

### Mortality

**FIGURE 1:**
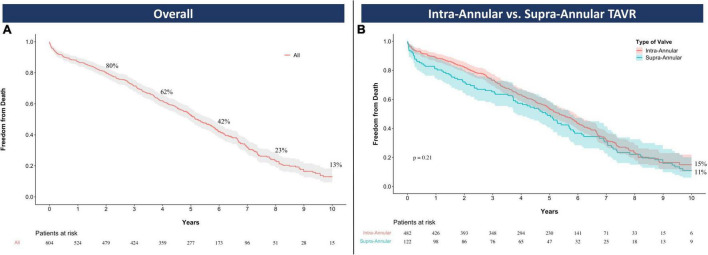
Overall survival with Kaplan Meier estimates for the entire study population **(A)** and the comparison between intra-annular and supra-annular devices **(B)**. TAVR, transcatheter aortic valve replacement.

Clinical follow-up was available for all the patients with a median duration of 4.9 years [IQR: 2.5–6.2] up to a maximum of 12.4 years. Sixty patients (10%) were lost after the 5-year follow-up. Four hundred and nine deaths were observed through the overall follow-up period with a median survival time of 3.8 years [IQR: 1.6–5.6]. Kaplan Meier estimates of overall survival at 2, 4, 6, 8, and 10 years were 80% (95%CI: 77–83), 62% (95%CI: 58–66), 42% (95%CI: 38–46), 23% (95%CI: 19–28), and 13% (95%CI: 9–18), respectively, Figure 1A. Stratifying the study population per TAVR leaflet design, there was no significant difference in 10-year survival between the IA (15%, 95%CI: 10–22) and the SA group (11%, 95%CI: 6–20, log rank *p* = 0.21) up to 10 years of follow-up, Figure 1B.

### Echocardiographic Findings

**FIGURE 2:**
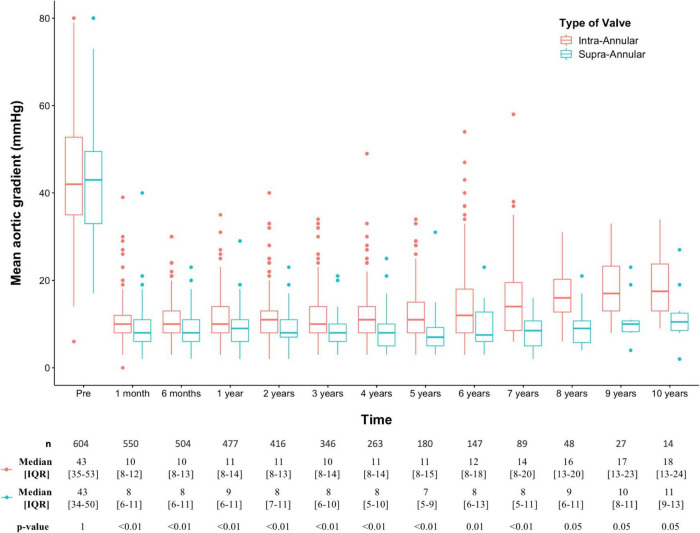
Pairwise analysis of intra-annular versus supra-annular transaortic mean gradients at each follow-up time; *p* values were adjusted using the Bonferroni multiple testing correction method and obtained with Wilcoxon rank sum test. IQR, interquartile range; SD, standard deviation.

All the collected echocardiographic data were grouped in the corresponding time points after TAVR. Each of these was individually analyzed stratifying for the IA and the SA group. At 30 days, there was an overall prevalence of 1.3% (IA 1.6%, SA 0%) for severe and 16% (IA 17.7%, SA 8.9%) for moderate prosthesis-patient mismatch. Pairwise comparisons showed significantly higher mean aortic gradients for the IA TAVRs at each time point assessed after TAVR (all *p* < 0.05). This finding was tested under different distribution assumptions and remained consistent whether pairwise analyses were performed using the Student’s *t* test (Supplementary Figure 1) or the Wilcoxon rank sum test (Figure 2).

Diverging trends in the evolution of mean transaortic gradients can be visually assessed in Supplementary Figures 1, 2. To better investigate these potential differences emerging over time, the 1-month post-TAVR echocardiographic evaluation was compared with every other time point using paired analyses. While the hemodynamic performance of SA devices was preserved throughout the entire follow-up period (*p* > 0.05), IA bioprostheses showed increasing transaortic gradients starting from the 1-year assessment post-TAVR (*p* < 0.05), Table 2 and Supplementary Table 2.

**TABLE 2:** Paired analysis of mean aortic gradients.

	Mean aortic gradient (mmHg)																	
	**30 days**			**6 months**			**1 year**			**2 years**			**3 years**			**4 years**		
	**Baseline**	**30 days**	***p*-value**	**30 days**	**6 months**	***p*-value**	**30 days**	**1 year**	***p*-value**	**30 days**	**2 years**	***p*-value**	**30 days**	**3 years**	***p*-value**	**30 days**	**4 years**	***p*-value**
Total	43 (35–53)	9 (7–12)	<0.01	10 (8–12)	10 (7–12)	0.3	10 (8–12)	10 (8–13)	<0.01	10 (8–12)	10 (8–13)	<0.01	9 (7–12)	10 (8–1)	<0.01	9 (7–12)	10 (8–14)	<0.01
IA	43 (35–53)	10 (8–12)	<0.01	10 (8–12)	10 (8–13)	0.2	10 (8–12)	11 (8–14)	<0.01	10 (8–12)	11 (8–13)	<0.01	10 (8–12)	10 (8–14)	<0.01	9 (8–12)	11 (8–14)	<0.01
SA	43 (34–50)	8 (6–11)	<0.01	9 (6–11)	8 (6–11)	1	9 (6–11)	9 (6–11)	0.6	9 (6–11)	8 (7–11)	0.9	8 (5–10)	8 (6–10)	0.6	9 (6–11)	8 (5–10)	0.3

	**5 years**			**6 years**			**7 years**			**8 years**			**9 years**			**10 years**		
	**30 days**	**5 years**	***p*-value**	**30 days**	**6 years**	***p*-value**	**30 days**	**7 years**	***p*-value**	**30 days**	**8 years**	***p*-value**	**30 days**	**9 years**	***p*-value**	**30 days**	**10 years**	***p*-value**

Total	9 (7–12)	10 (7–14)	<0.01	9 (7–12)	12 (8–16)	<0.01	9 (8–11)	10 (7–17)	<0.01	9 (8–12)	12 (8–14)	0.01	9 (8–12)	12 (9–19)	0.03	9 (8–12)	14 (10–19)	0.02
IA	9 (8–12)	11 (8–15)	<0.01	9 (8–12)	12 (8–18)	<0.01	8 (8–11)	14 (8–20)	<0.01	8 (8–12)	16 (13–20)	0.04	8 (8–12)	17 (13–23)	0.02	8 (8–12)	18 (13–24)	0.01
SA	8 (5–10)	7 (5–9)	0.3	9 (6–11)	8 (7–12)	0.5	9 (5–11)	8 (5–11)	0.3	10 (9–12)	9 (6–11)	0.6	10 (9–12)	11 (8–11)	1	10 (9–12)	12 (8–12)	0.8

*Paired analysis performed with the Wilcoxon signed rank-sum test. IA, intra-annular; SA, supra-annular.*

### Valve Durability at 10 Years

**FIGURE 3:**
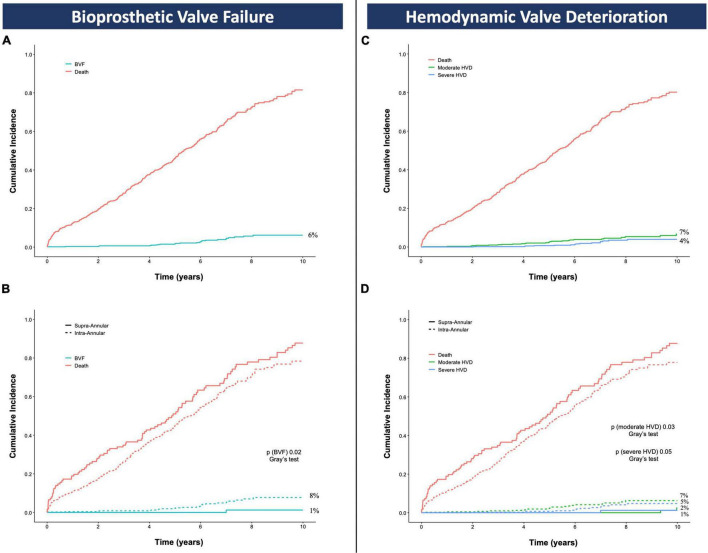
Cumulative incidence function of bioprosthetic valve failure **(A,B)** and hemodynamic valve deterioration **(C,D)** accounting for death as competing risk; *p* values are obtained with the Gray’s test and refer to the comparison between the intra-annular and the supra-annular group. BVF, bioprosthetic valve failure; HVD, hemodynamic valve deterioration.

Assuming death as a competing risk that can prevent the investigated outcome to happen, the 10-year CIF of BVF was 6% (*n* = 25) and was significantly higher in the IA compared to the SA group (8% [*n* = 24] vs. 1% [*n* = 1], Gray’s test *p* = 0.02), Figures 3A,B. The overall 10-year CIFs of moderate and severe HVD were 7% (*n* = 27) and 4% (*n* = 15), respectively, with the same trend for increased risk of the IA vs. the SA cohort (moderate HVD: 7% [*n* = 25] vs. 2% [*n* = 2], Gray’s test *p* = 0.03; severe HVD: 5% [*n* = 14] vs. 1% [*n* = 1], Gray’s test *p* = 0.05), Figures 3C,D.

Further details of the BVF events adjudicated throughout the overall follow-up period are described in Table 3. Of the 26 patients meeting the criteria for BVF, only 2 received a SA TAVR and experienced the event at 8.2 and 12.4 years after TAVR. In the IA group, 21 were balloon-expandable and 3 mechanically expandable devices; most common defining criteria for BVF were severe HVD (*n* = 20, 15 aortic stenosis, 4 aortic regurgitation, 1 combined aortic stenosis and regurgitation) and valve-related death (*n* = 10). The majority of valve failures were found in those patients who received the smallest bioprosthesis sizes (≤23-mm) and all of them experienced HVD. Two patients were re-intervened: one with a successful transcatheter valve-in-valve procedure, and the other with open heart surgery complicated by in-hospital death; one patient died during pre-procedural evaluation for re-intervention; three patients refused or were deemed not suitable for further procedures due to their poor functional status.

**TABLE 3:** Bioprosthetic valve failure.

No.	Age	Device, size	Access	Reason BVF	Time BVF (days)	Death	Last FU (days)	Clinical status
1.	82	Sapien XT, 23 mm	TA	Severe HVD (AS)	743	1	2,680	Refused ViV Poor-Performance Status
2.	67	Sapien XT, 23 mm	TF	Severe HVD (AR), LV dysfunction	1,485	1	1,721	Dead
3.	75	Sapien XT, 23 mm	TF	SVD, Moderate HVD (AS), Valve-related Death	1,554	1	1,554	Dead
4.	78	Sapien XT, 23 mm	TA	SVD, Moderate HVD (AS), NSVD, Symptoms	1,834	1	2,031	Dead
5.	82	Sapien XT, 23 mm	TA	SVD, Severe HVD (AS)	1,896	1	2,026	Dead
6.	77	Sapien XT, 23 mm	TF	Severe HVD (AS), ViV TAVR	2,133	0	2,149	Alive: NYHA III
7.	82	Sapien XT, 23 mm	TF	Severe HVD (AS + AR), Valve-related Death	2,190	1	2,438	Dead
8.	77	Sapien XT, 23 mm	TF	Severe HVD (AS)	2,205	0	2,892	Alive: NYHA II
9.	80	Sapien XT, 23 mm	TF	Severe HVD (AS)	2,589	1	3,223	Refused ViV Poor-Performance Status
10.	76	Sapien XT, 23 mm	TA	Severe HVD (AS), Valve-related Death	2,674	1	2,681	Dead
11.	79	Sapien XT, 23 mm	TF	Moderate HVD (AS), Valve-related Death	2,805	1	2,805	Dead
12.	85	Sapien XT, 23 mm	TF	Severe HVD (AS)	2,941	0	3,954	Alive: NYHA II
13.	84	Sapien XT, 26 mm	TA	SVD, Severe HVD (AR), LV dilation	1,470	1	1,613	Dead
14.	87	Sapien XT, 26 mm	TA	SVD, Severe HVD (AS), LV dysfunction	1,821	1	2,545	Dead
15.	74	Sapien XT, 26 mm	TF	Thrombosis, Endocarditis, SAVR, Valve-related Death	2,174	1	2,174	Dead after unsuccessful SAVR
16.	81	Sapien XT, 26 mm	TF	Moderate HVD (AR), LV dilation, Valve-related Death	2,208	1	2,208	Dead
17.	75	Sapien XT, 26 mm	TF	Severe HVD (AS)	2,262	0	2,632	Alive: NYHA II
18.	67	Sapien XT, 26 mm	TA	Severe HVD (AR), Valve-related Death	2,432	1	2,439	Dead
19.	77	Sapien XT, 26 mm	TF	Severe HVD (AS)	2,556	0	2,666	Alive: NYHA I
20.	86	Sapien 3, 26 mm	TA	Thrombosis, Valve-related Death	0	1	0	Dead
21.	84	Sapien 3, 29 mm	TF	NSVD, LV dysfunction	734	1	1,061	Dead
22.	86	Lotus, 23 mm	TF	Endocarditis, Valve-related Death	259	1	259	Dead
23.	70	Lotus, 23 mm	TF	Moderate HVD (AS), LV dysfunction	1,590	0	2,359	Alive: NYHA II-III
24.	76	Lotus, 25 mm	TF	SVD, Severe HVD (AS)	1,611	1	1,733	Dead during ViV evaluation
25.	73	CoreValve, 26 mm	TF	Severe HVD (AS + AR), Valve-related Death	4,379	1	4,516	Dead
26.	85	CoreValve, 29 mm	TF	Severe HVD (AS + AR)	2,992	1	2,992	Refused ViV Poor-Performance Status

*AR, aortic regurgitation; AS, aortic stenosis; BVF, bioprosthetic valve failure; FU, follow-up; HVD, hemodynamic valve deterioration; LV, left ventricular; NSVD, non-structural valve deterioration; NYHA, New York Heart Association; SAVR, surgical aortic valve replacement; SVD, structural valve deterioration; TA, transapical; TAVR, transcatheter aortic valve replacement; TF, transfemoral; ViV, valve-in-valve.*

**FIGURE 4:**
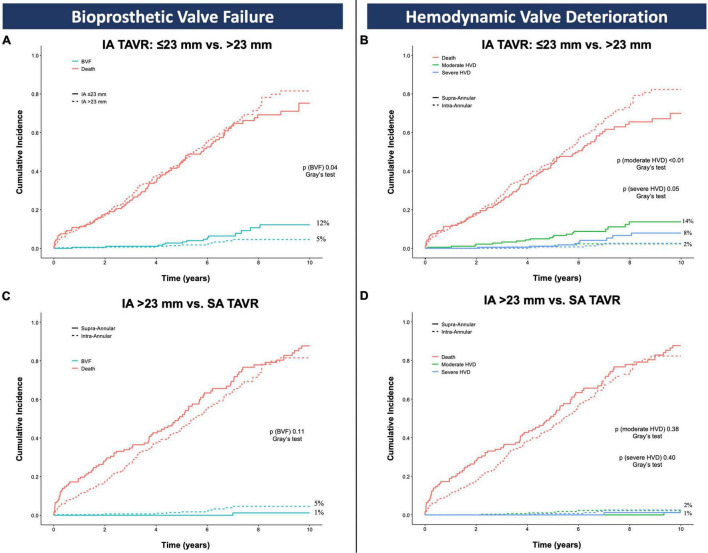
Cumulative incidence function of bioprosthetic valve failure **(A,C)** and hemodynamic valve deterioration **(B,D)** accounting for death as competing risk; *p* values are obtained with the Gray’s test and refer to the comparison between IA ≤ 23 mm vs. IA > 23 mm **(A,B)** and IA > 23 mm vs. SA group **(C,D)**. BVF, bioprosthetic valve failure; HVD, hemodynamic valve deterioration; IA, intra-annular; SA, supra-annular; TAVR, transcatheter aortic valve replacement.

As sensitivity analysis, CIF of BVF was assessed after exclusion of the LOTUS devices with similar results compared to the main analysis on the overall population (BVF IA 7% vs. SA 1%, Gray’s test *p* = 0.03), Supplementary Figure 2. Also, CIF of BVF and HVD were assessed in the IA group comparing the ≤ 23 mm vs. >23 mm devices. In the IA group, CIF of BVF (IA < 23 mm 12% vs. IA > 23 mm 5%, Gray’s test *p* = 0.04), moderate HVD (IA < 23 mm 14% vs. IA > 23 mm 2%, Gray’s test *p* < 0.01), and severe HVD (IA < 23 mm 8% vs. IA > 23 mm 2%, Gray’s test *p* = 0.05) were mainly driven by the smallest (≤23 mm) valve sizes, Figures 4A–C. After excluding the IA ≤ 23 mm valves, CIF of BVF (IA 5% vs. SA 1%, Gray’s test *p* = 0.11), moderate HVD (IA 2% vs. SA 2%, Gray’s test *p* = 0.38), and severe HVD (2% vs. 1%, Gray’s test *p* = 0.40) showed a similar trend compared to the analyses performed on the overall population with no significant differences in each of these VARC-3 outcomes per TAVR leaflet design, Figures 4B–D.

## Discussion

As TAVR indication continues to expand to a population with longer life expectancy, device durability and management of long-term device failures are taking central stage in clinical research and training. The present study provides the largest head-to-head comparison of IA versus SA TAVRs beyond the landmark time of 5 years post-procedure. The main findings of this 10-year analysis can be summarized as follows: (1) overall survival was not affected by TAVR leaflet design; (2) IA leaflet design results in higher mean aortic gradients and cumulative incidence of BVF and HVD; and (3) no significant differences in BVF and HVD were evident after exclusion of the ≤ 23-mm IA valve sizes, Graphical Abstract.

In terms of all-cause mortality, we found no significant differences between the IA and the SA group. This finding is consistent with previous publications reporting a survival after TAVR of 23.2% at 7 years (9), and 22–27% at 8 years (10, 11). Our results extend the 5-year analysis of the CHOICE randomized trial (4), suggesting that long-term mortality is largely determined by advanced age and comorbid conditions rather than the TAVR leaflet design. Accordingly, it is not surprising to observe an overall survival as low as 23 and 13%, at 8 and 10 years, respectively.

With regards to hemodynamic performance, a SA design was associated with more stable forward-flow hemodynamics throughout the follow-up period, consistent with previous long-term analysis investigating the CoreValve device (10, 11). On the contrary, previous studies on IA TAVRs showed conflicting results, some reporting stable (12, 13), other increasing (4, 14) transaortic gradients following TAVR. The findings of this study are in agreement with those by Abbas et al. that reported significantly higher gradients for IA devices by both invasive and echocardiographic measurements (14). In our study, transaortic gradients continued to increase progressively up to 10-years following initial TAVR implantation. Whether this trend might differ between the small vs. large sizes of IA valves needs further long-term analyses (14, 15).

Although the study period allowed us to investigate the performance of previous-generation devices in high/intermediate-risk patients, we can speculate that the observed hemodynamic differences might be similar with the current generation valves and/or in younger patients. In fact, data from the low-risk TAVR trials show that while new-generation SA devices continue to outperform surgical valves (16), novel IA bioprostheses were found to be inferior to surgical bioprostheses in terms of effective orifice area (17). The 10-year follow-up data of low-risk TAVR trials are eagerly awaited to provide us with definitive evidence on this topic.

The unclear impact of worsening hemodynamic performance on clinical outcomes led the European and American task forces to define standardized criteria for valve failure (8, 18). Our study is one of the first available analyses adopting the recently updated VARC-3 criteria. We found a 10-year cumulative incidence of BVF, moderate HVD, and severe HVD of 6, 7, and 4%, respectively. Despite a direct comparison with the historical surgical data being inappropriate due to heterogeneities in patients and outcome definitions, these low rates are reassuring and do not show safety concerns for TAVR candidates (19, 20). Reviewing previous studies in TAVR populations there are similar BVF rates at 6-year (7.5%) (21) and 8-year follow-up (4.5%, 2.5%) (10, 11, 22).

It is important to note that adverse outcomes were more commonly observed in the IA cohort. One SA device experienced a BVF in the 10-year period, with the majority of events observed for IA TAVRs and mainly driven by HVD. This finding is in line with their increased transaortic gradients leading to the occurrence of more moderate/severe HVDs. Although several factors can play a role in valve function (mode of crimping, mechanism of deployment, post-dilatation, frame expansion, and anti-calcification treatment), our data suggest that hemodynamic performance is a major determinant of long-term durability. SA TAVR results in higher indexed effective orifice area and lower mean aortic gradients compared to IA devices (23). Given that the majority of BVF in our series were caused by a stenotic HVD, the hemodynamic properties of a SA design might confer an advantage in terms of long-term valve durability. In the CHOICE randomized trial, the 5-year cumulative incidences of BVF were similar between IA (4.3%) and SA (3.4%) devices, while the transaortic gradients and the rates of HVD were clearly in favor of SA TAVRs (0% vs. 6.6%, Gray’s test *p* = 0.018) (4). Interestingly, the curves of aortic gradients and HVD started diverging just before the 5 years. Even though a longer follow-up seems unfeasible due to the limited numbers of CHOICE patients alive at 5 years (IA = 46, SA = 42), we believe that by extending this observation time, the reported differences would have been even broader with direct consequences on the “harder” endpoint of BVF. Indeed, similarly to surgical bioprostheses, the CIF curves of the present study show that the critical period when most valve failure occur ranges from 5 to 10 years following TAVR. Besides TAVR leaflet design, patient-related factors have been suggested to increase (24) the incidence of BVF, particularly conditions leading to dysregulation of phosphocalcic metabolism, such chronic kidney disease. To note, in our study population baseline patient characteristics were well balanced between SA and IA group, with renal insufficiency being more present in the SA group.

*Post hoc* analysis of the pivotal trials comparing surgical and transcatheter therapies (namely PARTNER [Placement of Aortic *Trans-* catheter Valves] and the U.S. CoreValve High Risk Study) have shown lower rates of prosthesis-patient mismatch with TAVR than with surgical replacement (25, 26), regardless of the use of stentless or stented surgical valves (27). This improved hemodynamic performance seems to be even more pronounced in small anatomies (26). However, the degree of such benefit is not equivalent among all the TAVRs, and the hemodynamic outperformance of SA valves has been proved to be even more pronounced in small anatomies compared to IA devices (14, 15). A detailed look at our series of BVFs supports this concept, as a significant part of these patients received the smallest sizes of the IA bioprostheses (≤23 mm) and all of them experienced HVD. Excluding the smallest IA device sizes (≤23 mm), the differences in BVF and HVD per TAVR leaflet design were no longer evident. This finding is reassuring and underlines the importance of assessing the valve size in combination with its leaflet design when anticipating TAVR valve durability.

The choice of the most appropriate TAVR device for each type of patient is complex and requires the assessment of several factors. Amongst them, future coronary access, the need for subsequent percutaneous procedures (28), the risk of coronary obstruction in case of TAVR-in-TAVR (29, 30), and the risk for new pacemaker implantation (31, 32) are some important considerations to keep in mind that can favor one device over another. This study highlights the role of forward-flow hemodynamic on long-term valve durability and provides further insights on this multifactorial and patient-centered approach when choosing the TAVR device.

### Study Limitations

It has to be acknowledged that the study inclusion period (2007–2016) entails an initial learning curve and the use of old-generation TAVR devices. However, similar long-term analyses using modern implantation techniques and new-generation bioprostheses will not be available for several years. When analyzing a high-risk and old cohort of patients, such as those treated in the study period, it must be recognized that death represents an important competing risk. To limit this effect, we reported actual estimates of BVF and HVD using the CIF, as recommended by the European and American consensus statements (8, 18). The finding of a higher incidence of valve degeneration of IA vs. SA TAVRs and the absence of significant differences excluding the smallest IA valve sizes have to be considered hypothesis generating and needs to be confirmed by larger, randomized studies with long-term follow up. Unfortunately, such analyses will not be available in the near future.

## Conclusion

At 10 years after TAVR, survival was not affected by TAVR leaflet design. The hemodynamic performance of SA valves was maintained long term. Mean transaortic gradients of IA devices were significantly higher with a progressive increase over time. Cumulative incidences of HVD and BFV were low, with higher rates in the IA compared to the SA TAVRs. No differences in BVF and HVD were evident after exclusion of the smallest IA valve sizes. These rates of long-term degeneration of SA and IA devices need to be confirmed by larger randomized studies with long-term follow up.

## Data Availability Statement

The raw data supporting the conclusions of this article will be made available by the authors, without undue reservation.

## Ethics Statement

The studies involving human participants were reviewed and approved by the IRB of the University Hospital of Padua. The patients/participants provided their written informed consent to participate in this study.

## Author Contributions

AS, LF, AC, JG, AL, and GT participated to the conception, design, analysis, interpretation of data, and drafting of the manuscript. All authors contributed providing a critical revision for important intellectual content, giving the final approval of the submitted text, agreeing to be accountable for all aspects of the work in ensuring that questions related to the accuracy or integrity of any part of the work are appropriately investigated and resolved, and have contributed significantly to the submitted work.

## Conflict of Interest

GT reports honoraria for lectures/consulting from Medtronic, Edwards Lifesciences, Boston Scientific, and Abbott. AL is an advisor and reports honoraria for consulting from Medtronic, Edwards Lifesciences, Boston Scientific, and Abbott. The remaining authors declare that the research was conducted in the absence of any commercial or financial relationships that could be construed as a potential conflict of interest.

## Publisher’s Note

All claims expressed in this article are solely those of the authors and do not necessarily represent those of their affiliated organizations, or those of the publisher, the editors and the reviewers. Any product that may be evaluated in this article, or claim that may be made by its manufacturer, is not guaranteed or endorsed by the publisher.

## Abbreviations

BVF: bioprosthetic valve failure
HVD: hemodynamic valve deterioration
IA: intra-annular
SA: supra-annular
TAVR: transcatheter aortic valve replacement.
