# Supplementary material for: 10-Year Impact of Transcatheter Aortic Valve Replacement Leaflet Design (Intra- Versus Supra-Annular) in Mortality and Hemodynamic Performance
Source: Front Cardiovasc Med. 2022 Jun 8;9:924958. doi: 10.3389/fcvm.2022.924958 (PMC9215259; doi:10.3389/fcvm.2022.924958)

Supplementary Material

# Supplementary Table S1. TAVR Devices

| TAVR Device | IA (482) | SA (122) |
| --- | --- | --- |
| Sapien XT | **230 (48)** |  |
| 23 mm | 89 |  |
| 26 mm | 114 |  |
| 29 mm | 27 |  |
| Sapien 3 | **195 (40)** |  |
| 20 mm | 1 |  |
| 23 mm | 69 |  |
| 26 mm | 87 |  |
| 29 mm | 38 |  |
| Lotus | **57 (12)** |  |
| 23 mm | 26 |  |
| 25 mm | 17 |  |
| 27 mm | 14 |  |
| CoreValve / Evolut R |  | **99 (81)** |
| 23 mm |  | 1 |
| 26 mm |  | 48 |
| 29 mm |  | 50 |
| Acurate |  | **23 (19)** |
| 23 mm |  | 7 |
| 25 mm |  | 10 |
| 27 mm |  | 6 |

IA: intra-annular; SA: supra-annular; TAVR: Transcatheter Aortic Valve Replacement

# Supplementary Table S2. Paired analysis of mean aortic gradients

|  | **Mean Aortic Gradient (mmHg)** | | | | | | | | | | | | | | | | | | |
| --- | --- | --- | --- | --- | --- | --- | --- | --- | --- | --- | --- | --- | --- | --- | --- | --- | --- | --- | --- |
|  | **30 days** | | | **6 months** | | | **1 year** | | | **2 years** | | | **3 years** | | | **4 years** | | | |
|  | **Baseline** | **30 days** | ***p value*** | **30 days** | **6 months** | ***p value*** | **30 days** | **1 year** | ***p value*** | **30 days** | **2 years** | ***p value*** | **30 days** | **3 years** | ***p value*** | **30 days** | **4 years** | | ***p value*** |
| **Total** | 45±15 | 10±4 | <0.01 | 10±4 | 10±4 | 0.6 | 10±4 | 11±5 | <0.01 | 10±4 | 11±5 | <0.01 | 10±4 | 11±5 | <0.01 | 10±4 | 10±6 | | <0.01 |
| **IA** | 45±15 | 10±4 | <0.01 | 10±4 | 10±4 | 0.4 | 10±4 | 11±4 | <0.01 | 10±4 | 11±5 | <0.01 | 10±4 | 12±5 | <0.01 | 10±4 | 12±6 | | <0.01 |
| **SA** | 45±16 | 9±5 | <0.01 | 9±5 | 9±4 | 0.7 | 9±5 | 9±4 | 0.4 | 9±5 | 9±4 | 0.9 | 9±5 | 8±4 | 1 | 9±5 | 8±3 | | 0.2 |
|  | **5 years** | | | **6 years** | | | **7 years** | | | **8 years** | | | **9 years** | | | **10 years** | | | |
|  | **30 days** | **5 years** | ***p value*** | **30 days** | **6 years** | ***p value*** | **30 days** | **7 years** | ***p value*** | **30 days** | **8 years** | ***p value*** | **30 days** | **9 years** | ***p value*** | **30 days** | | **10 years** | ***p value*** |
| **Total** | 10±5 | 12±6 | <0.01 | 10±5 | 14±10 | <0.01 | 10±3 | 14±10 | <0.01 | 10±3 | 14±8 | 0.05 | 10±3 | 15±8 | 0.01 | 10±3 | | 16±8 | 0.01 |
| **IA** | 10±4 | 12±6 | <0.01 | 10±4 | 15±10 | <0.01 | 10±3 | 16±11 | <0.01 | 10±4 | 17±8 | 0.02 | 10±4 | 19±9 | 0.01 | 10±4 | | 19±9 | 0.01 |
| **SA** | 9±6 | 8±4 | 0.2 | 11±8 | 9±5 | 0.4 | 10±3 | 8±4 | 0.2 | 10±2 | 11±5 | 0.9 | 10±3 | 11±6 | 0.6 | 10±3 | | 12±7 | 0.5 |

Paired analysis performed with the Student’s paired t-test. IA: intra-annular; SA: supra-annular.

**Supplementary Figure 1.** Pairwise analysis of intra-annular versus supra-annular transaortic mean gradients at each follow-up time; p values were adjusted using the Bonferroni multiple testing correction method and obtained with Student’s t test. IQR: interquartile range; SD: standard deviation.


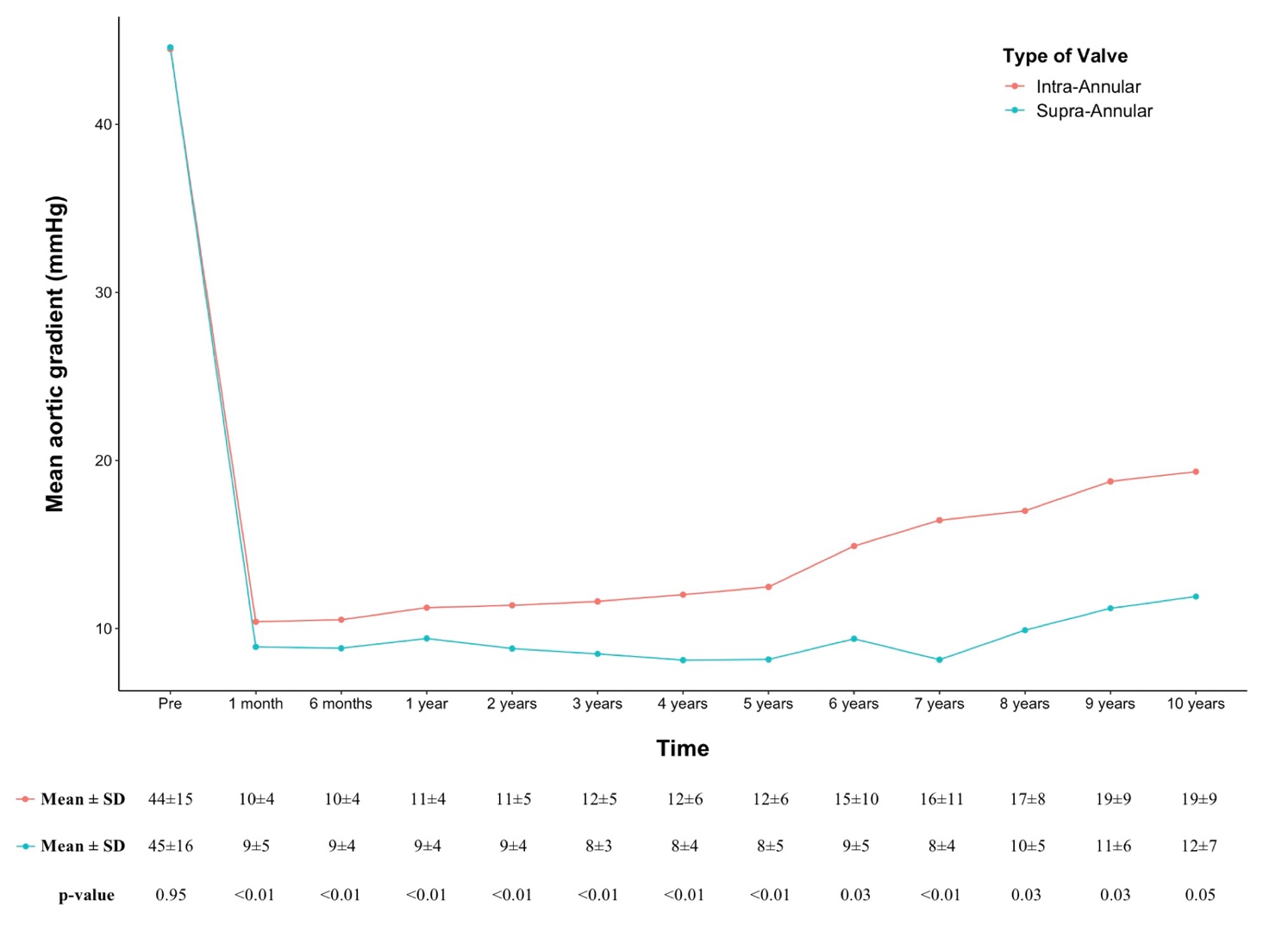


**Supplementary Figure 2**. Exclusion of TAVR with LOTUS valves. Cumulative incidence function of Bioprosthetic Valve Failure accounting for death as competing risk; p values are obtained with the Gray’s test and refer to the comparison between the intra-annular and the supra-annular group. BVF: Bioprosthetic Valve Failure.


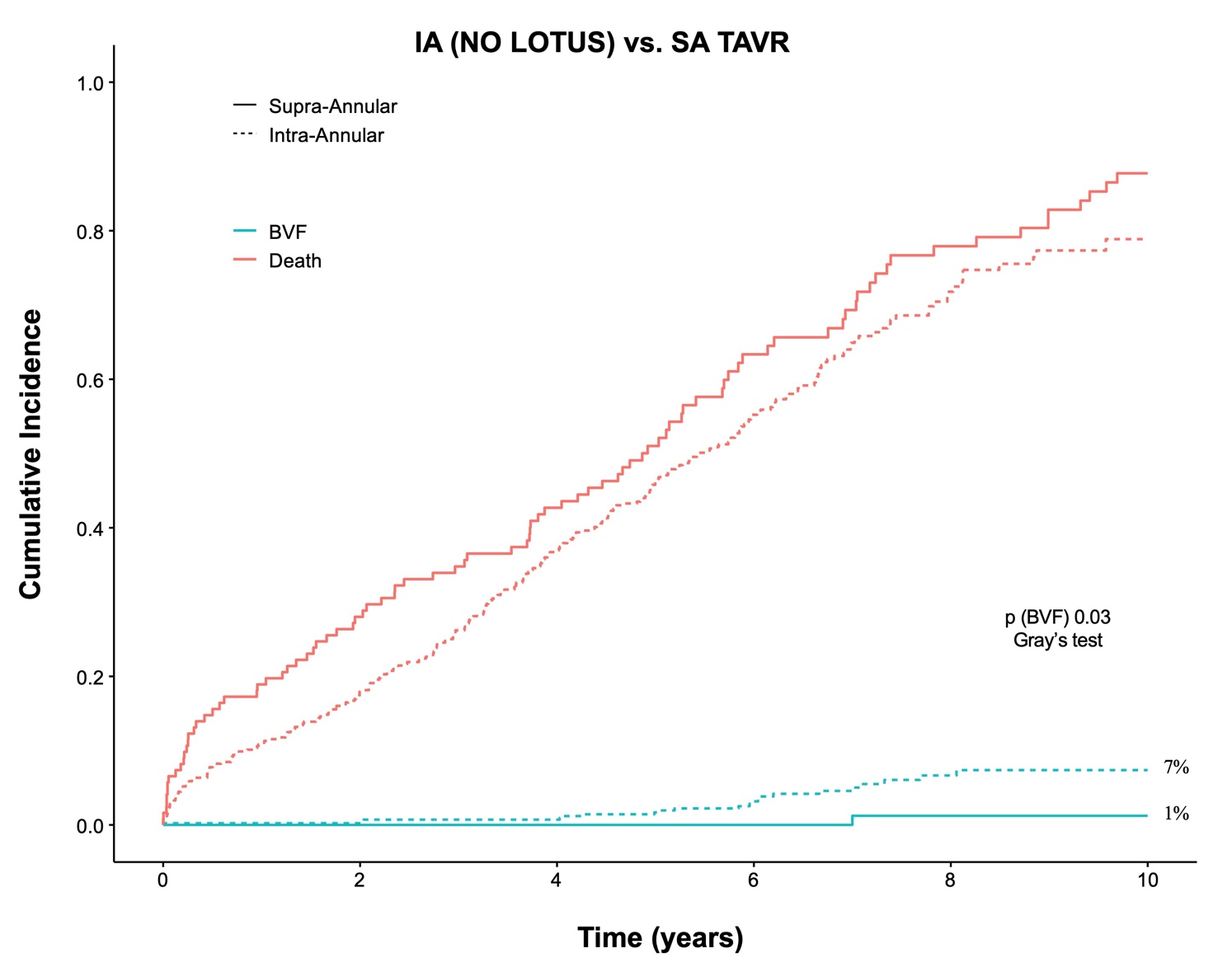

Supplement: Supplementary file 1 [file Data_Sheet_1.docx]
